# Supplementary figures and images for: The piRNA protein Asz1 is essential for germ cell and gonad development in zebrafish and exhibits differential necessities in distinct types of germ granules
Source: PLoS Genet. 2025 Jan 13;21(1):e1010868. doi: 10.1371/journal.pgen.1010868 (PMC11760641; doi:10.1371/journal.pgen.1010868)

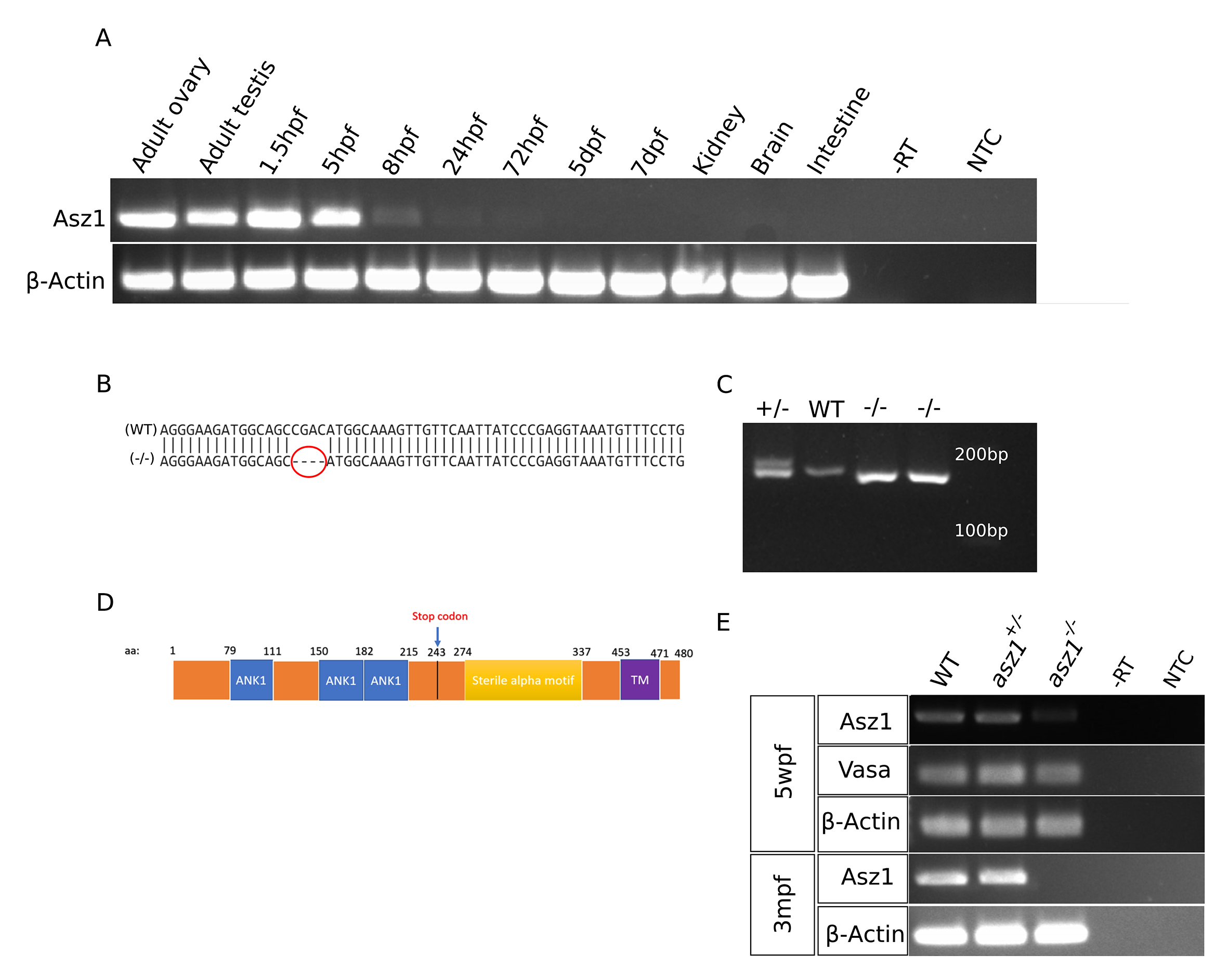

Supplement: S1 Fig — A. RT-PCR analysis showing specific expression of asz1 in ovaries and testes, with maternal deposition in early embryos, and detection of likely zygotic transcripts in 8 and 24 hpf embryos. -RT – no reverse transcriptase control; NTC – no DNA template control. B. wt and asz1huj102 genomic sequence (from Sanger sequencing) showing a 4 bp deletion in asz1huj102, leading to premature Stop codon at amino acid position 243 (D). C. PCR analysis of genomic DNA from wt, asz1+/huj102 and asz1huj102/huji102 fish, followed by Metapore gel electrophoresis (with 2% electrophoresis resolution), showing distinct bands of the asz1 locus in the three genotypes (individual fish per genotype; two homozygous fish are shown). Amplicons are 170 and 166 bp in the wt and asz1huj102 alleles, respectively. D. A scheme of the zebrafish Asz1 protein, showing the Ankyrin repeats, Sterile alpha motif, and transmembrane domain. The top arrow indicates the position of the premature STOP codon at amino acid position 243 in the asz1huj102 allele. E. RT-PCR analysis of asz1 expression in wt, asz1+/huj102 and asz1huj102/huji102 gonads at 5 wpf (n = 50 gonads per genotype) and 3 mpf (n = 6 gonads per genotype). β-act expression serves as loading control. Ddx4 expression marks the presence of germ cells at 5 wpf. asz1 transcripts are not detected in 3 mpf gonads. At 5 wpf, asz1 transcripts are only very weakly detected, despite vasa expression, demonstrating the likely decay of the asz1 mRNA by nonsense mediated decay. -RT – no reverse transcriptase control; NTC – no DNA template control. (TIFF) [file pgen.1010868.s002.tiff]

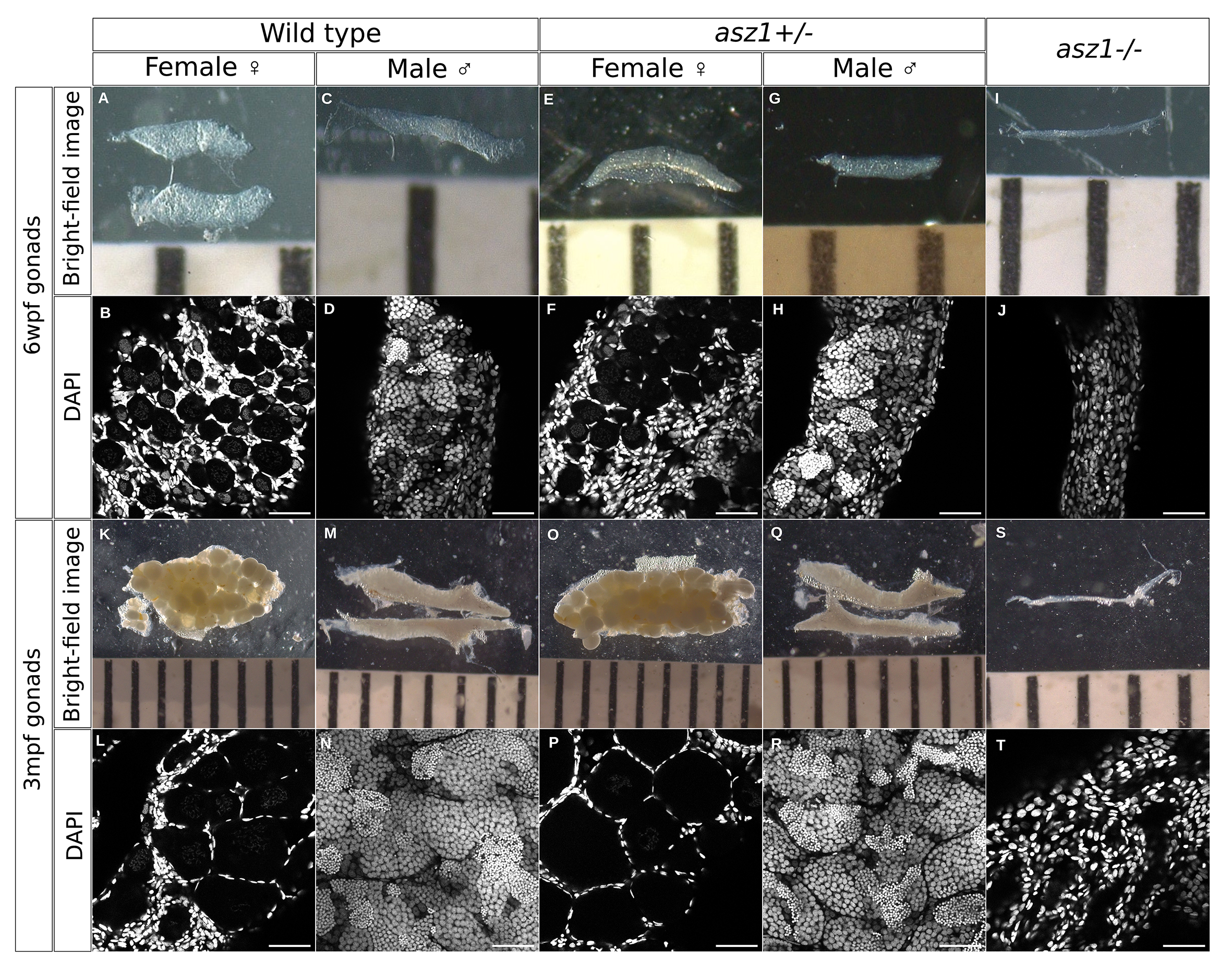

Supplement: S2 Fig — A, C, E, G, I. Representative brightfield images of wt, asz1+/-, or asz1-/- gonads at 6 wpf. Ruler grades are 1 mm. B, D, F, H, J. Confocal images of the same gonads from the brightfield images above, labeled for DAPI (greyscale). Wt and asz1+/- gonads exhibited normal developing oocytes and early spermatocytes in ovaries and testes as indicated, as well as oogonia and spermatogonia as generally detected by germ and somatic cell nuclear morphology (DAPI). asz1-/- gonads were much thinner with no clear detection of presumptive germ cells. Scale bars are 50 μm. K, M, O, Q, S. Representative brightfield images of adult wt, asz1+/-, or asz1-/- gonads at 3 mpf. Ruler grades are 1 mm. L, N, P, R, T. Confocal images of the same adult gonads from the brightfield images above, labeled for DAPI (greyscale). Wt and asz1+/- gonads exhibited normal ovarian and testes morphology as generally detected by DAPI (oocyte show weaker DAPI signal than their surrounding follicle cells), while asz1-/- gonads were much thinner with no clear detection of presumptive germ cells, and exhibited gaps in the tissue. Scale bars are 50 μm. (TIFF) [file pgen.1010868.s003.tiff]

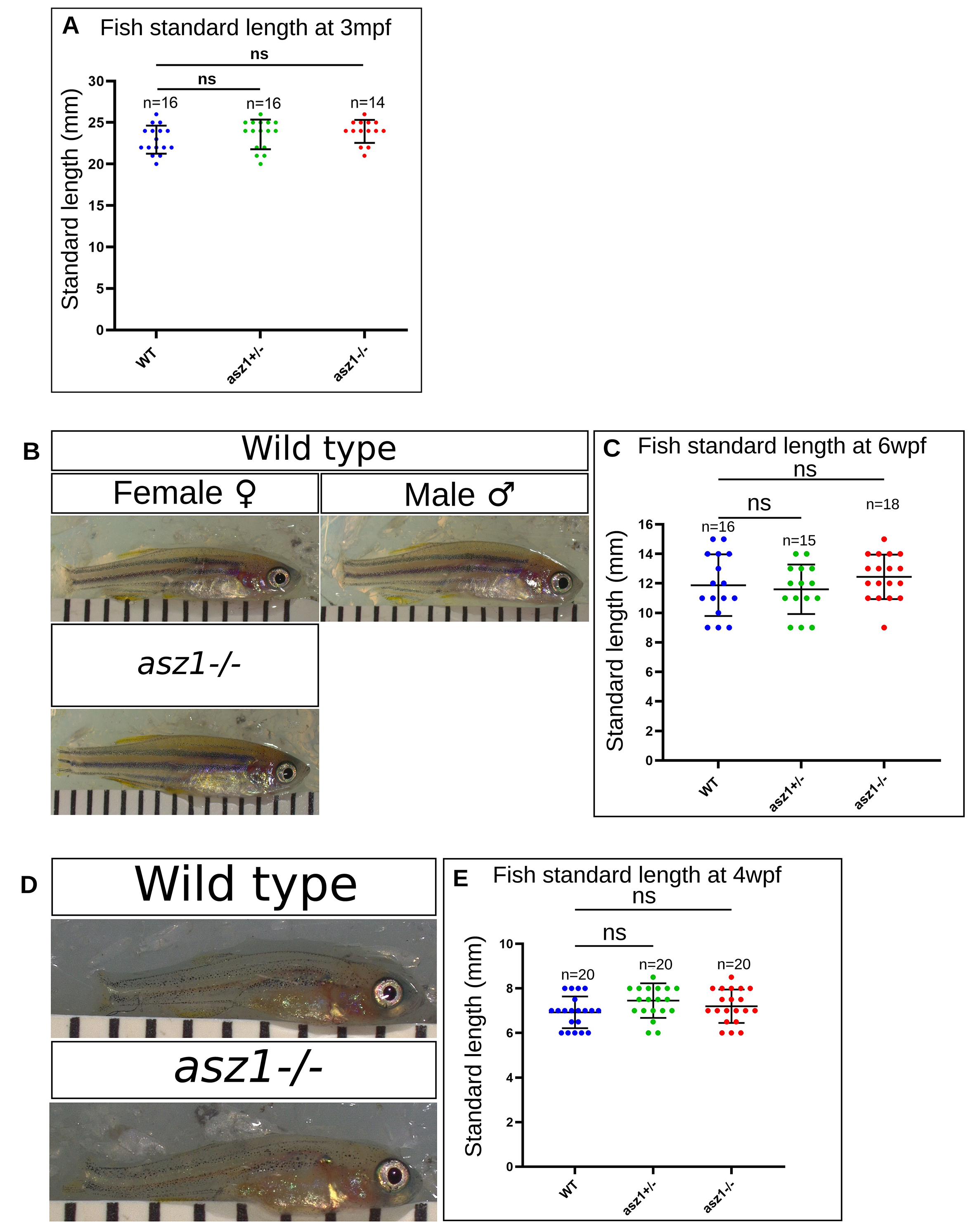

Supplement: S3 Fig — A. A plot of the SL of fish from all genotypes from Fig 1. n = number of fish. SL was not significantly different between genotypes. Bars are mean ± standard deviation (SD). B-C. Images of juvenile fish at 6 wpf from Fig 2 (B) and their SL (C). D-E. Images of juvenile fish at 4 wpf from Fig 3 (D) and their SL (E). In all panels, n = number of fish. SL was not significantly different between genotypes. Bars are mean ± standard deviation (SD). (TIFF) [file pgen.1010868.s004.tiff]

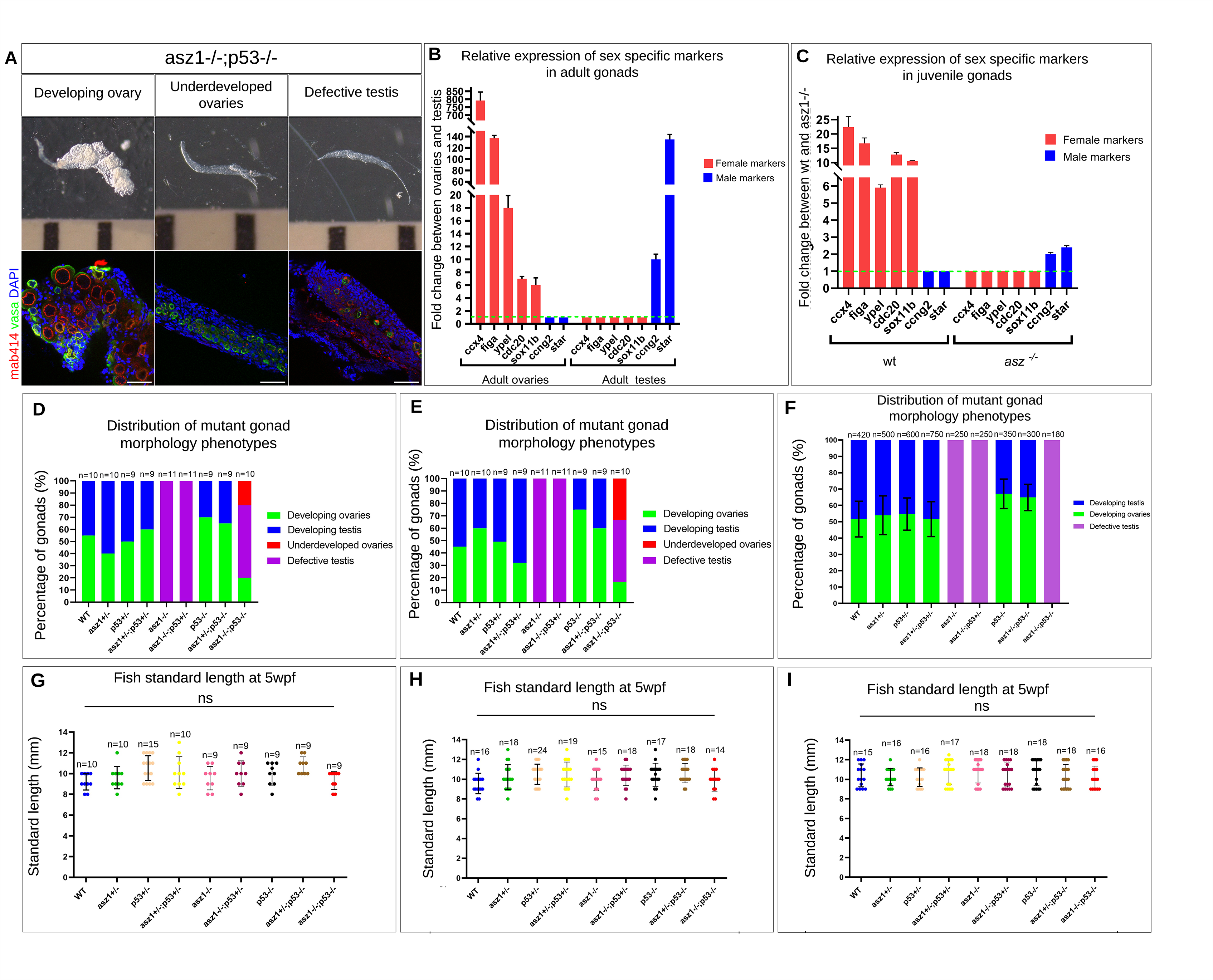

Supplement: S4 Fig — A. Representative images of the gonad categories in asz1-/-;tp53-/- fish. Top panels are brightfield images of the gonads in the bottom panels which are labeled with Ddx4 (green), mAb414 (red), and DAPI (blue). Scale bars are 50 μm. Developing ovaries are thicker, and contain oogonia and differentiating oocytes. Underdeveloped ovaries are thinner and contain oogonia with no further progressing oocytes. Defective testes are gonads that exhibit the asz1-/- phenotypes, being very thin and containing very few germ cells with abnormal mAb414 signal. These categories are consistent with our previous description of the morphology of normal and defective juvenile ovaries and testes [39]. The distribution of gonad categories per genotype are plotted for the two independent crosses that yielded ovaries (D-E). N = number of gonads. B. RT-qPCR of female-specific (red) and male-specific (blue) marker genes in adult ovaries and testes (B) confirm the sex-specific expression of those markers. Bars are ± SD between independent experiments. C. RT-qPCR on wt and asz1-/- juvenile gonads exhibit female-specific marker gene expression in wt, but reduced female-marker expression and elevated male-specific marker gene expression. Bars are ± SD between independent experiments. F. The distribution of gonad categories in all crosses that did not yield ovaries in asz1-/-;tp53-/- fish. A total of 17 rounds of in-crosses between 40 asz1+/-;tp53+/- double heterozygous individual fish were performed. In two crosses (panels D-E) a total of 6 asz1-/-;tp53-/- ovaries were obtained. In the remaining 15 crosses (panel F), all 180 asz1-/-;tp53-/- gonads were defective testes. n = number of gonads. Bars are mean ± SD between independent crosses. G-I. SL of fish from crosses in D-I, respectively. (TIFF) [file pgen.1010868.s005.tiff]

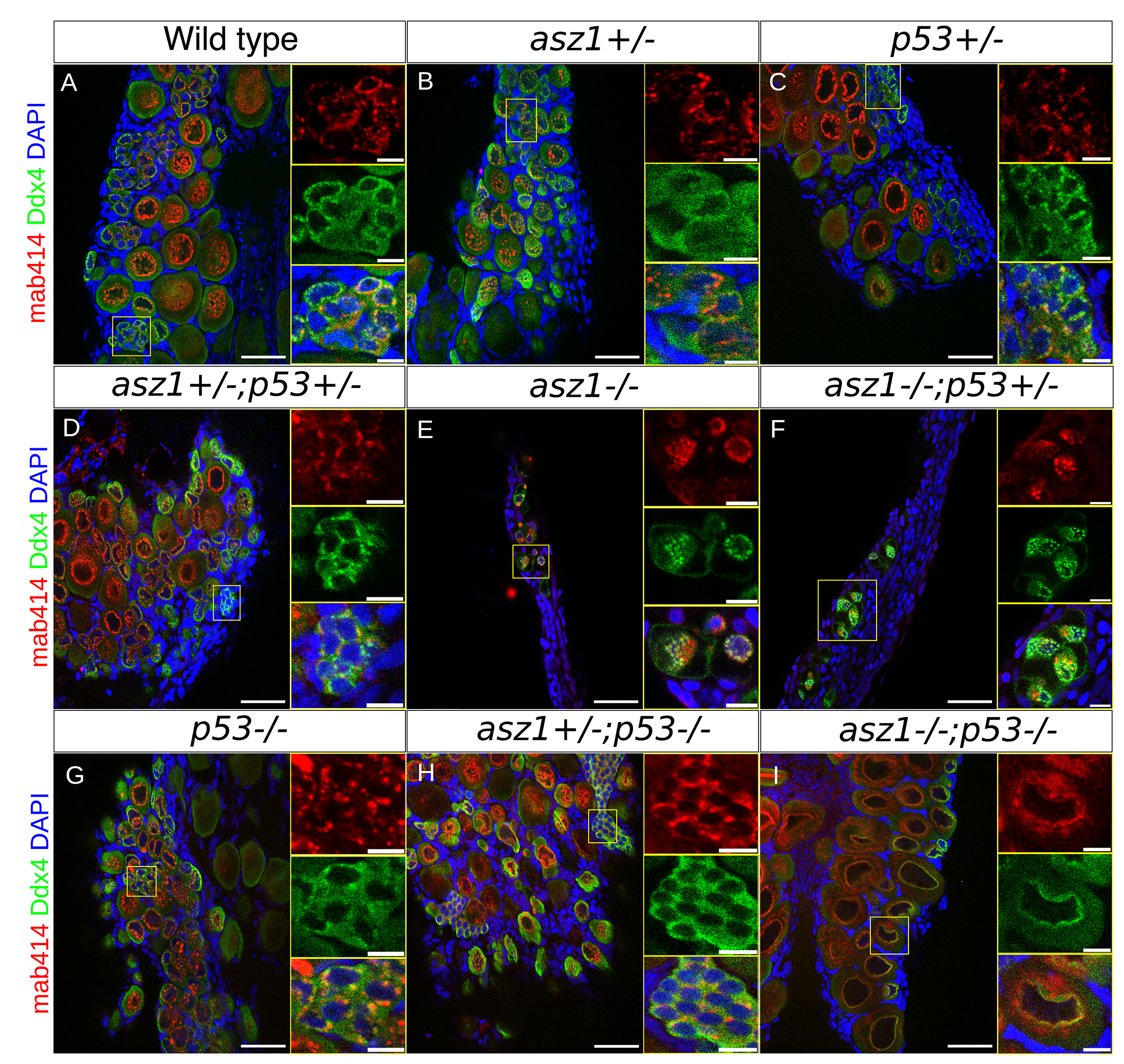

Supplement: S5 Fig — Overview of gonads of all asz1;tp53 genotypes from the experiment in Fig 6A–6I, labeled for Ddx4 (green), mAb414 (red), and DAPI (blue). Right panels are single and merged channel zoom-in magnifications of the yellow boxes in the overview panels. Scale bars are 50 μm and 10 μm in zoomed out and inset magnification images, respectively. (TIFF) [file pgen.1010868.s006.tiff]

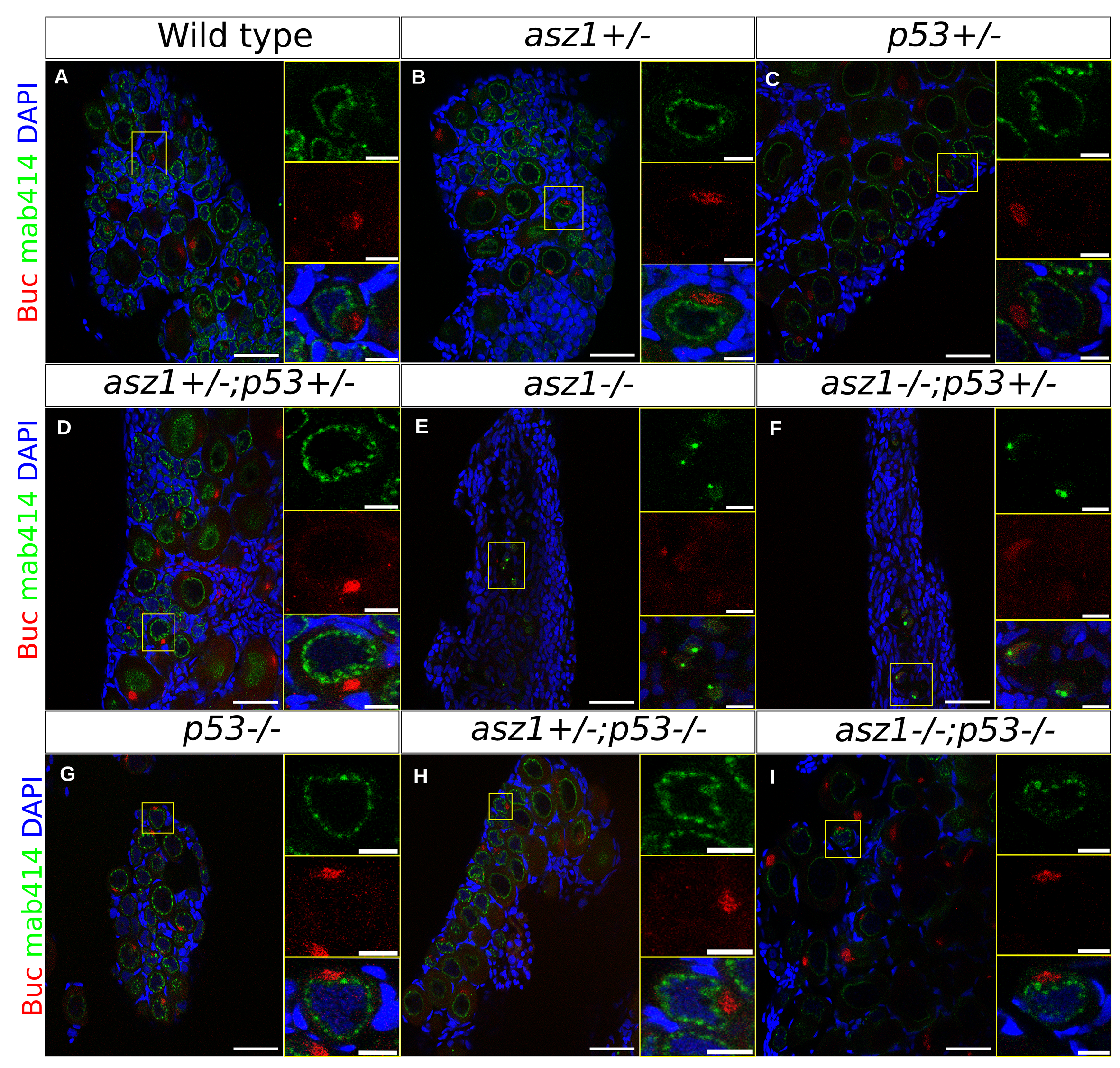

Supplement: S6 Fig — Overview of gonads of all asz1;tp53 genotypes from the experiment in Fig 6J and 6K, labeled for Buc (red), mAb414 (green), and DAPI (blue). Right panels are single and merged channel zoom-in magnifications of the yellow boxes in the overview panels. Scale bars are 50 μm and 10 μm in zoomed out and inset magnification images, respectively. All gonads (ovaries), show normal Buc localization in the forming Bb, except for the testes in asz1-/-, and asz1-/-;tp53+/-. n = 3-6 gonad per genotype, except 2 asz1-/-;tp53-/- ovaries. (TIFF) [file pgen.1010868.s007.tiff]

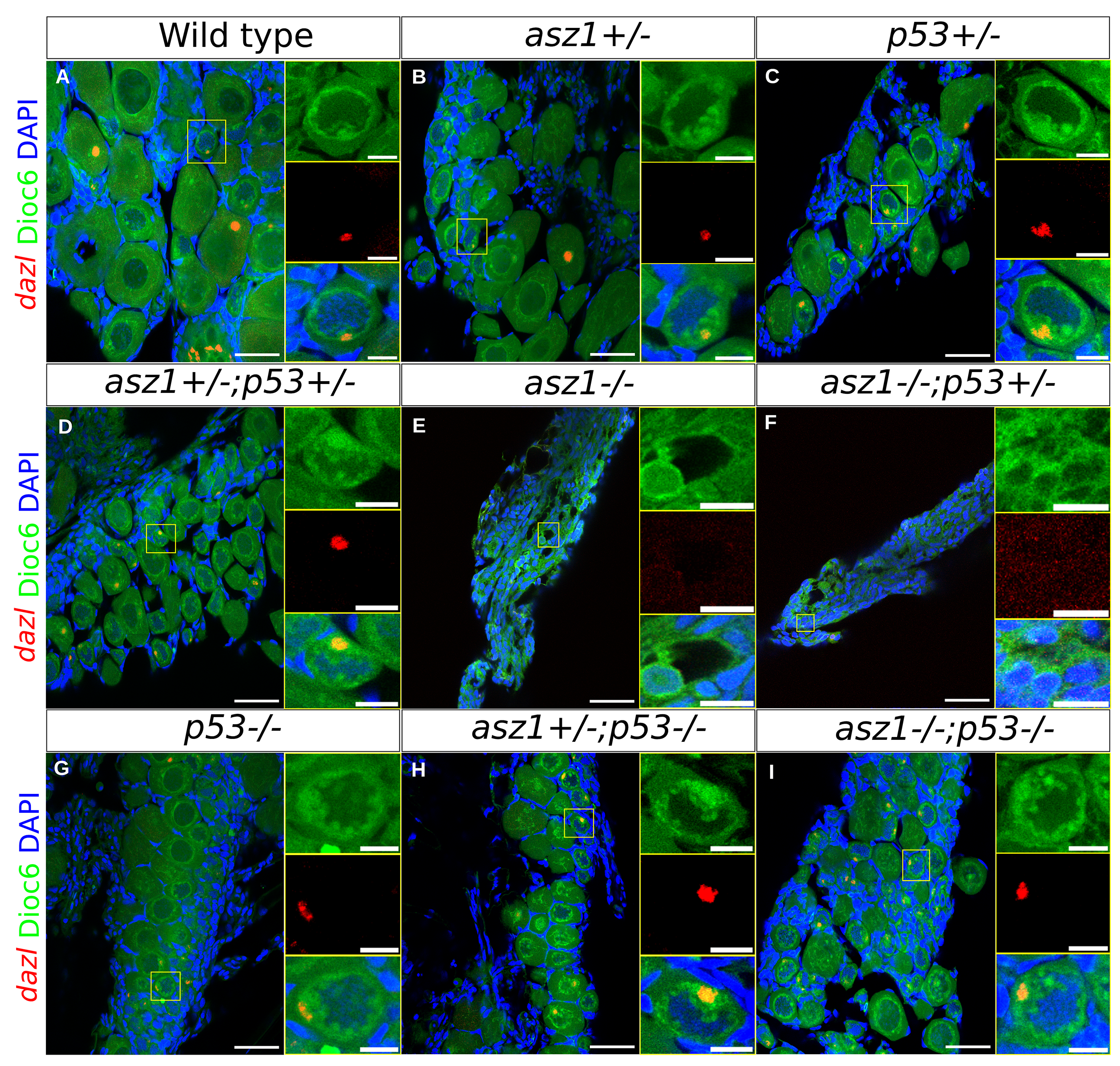

Supplement: S7 Fig — Overview of gonads of all asz1;tp53 genotypes from the experiment in Fig 6L and 6M, labeled for dazl (red), DiOC6 (green), and DAPI (blue). Right panels are single and merged channel zoom-in magnifications of the yellow boxes in the overview panels. Scale bars are 50 μm and 10 μm in zoomed out and inset magnification images, respectively. All gonads (ovaries), show normal dazl localization in the forming Bb, except for the testes in asz1-/-, and asz1-/-;tp53+/-. n = 3-5 gonad per genotype, except 2 asz1-/-;tp53-/- ovaries. (TIFF) [file pgen.1010868.s008.tiff]
